# lncRNA MIAT functions as a competing endogenous RNA to upregulate DAPK2 by sponging miR-22-3p in diabetic cardiomyopathy

**DOI:** 10.1038/cddis.2017.321

**Published:** 2017-07-13

**Authors:** Xiang Zhou, Wei Zhang, Mengchao Jin, Jianchang Chen, Weiting Xu, Xiangqing Kong

**Affiliations:** 1 Department of Cardiology, The Second Affiliated Hospital of Soochow University, Suzhou, China; 2 Department of Cardiology, The First Affiliated Hospital of Nanjing Medical University, Nanjing, China

## Abstract

We previously established a rat model of diabetic cardiomyopathy (DCM) and found that the expression of long non-coding RNA myocardial infarction–associated transcript (MIAT) was significantly upregulated. The present study was aimed to determine the pathologic role of MIAT in the development of DCM. MIAT knockdown was found to reduce cardiomyocyte apoptosis and improve left ventricular function in diabetic rats. High glucose could increase MIAT expression and induce apoptosis in cultured neonatal cardiomyocytes. The results of luciferase reporter assay and RNA immunoprecipitation assay revealed that MIAT was targeted by miR-22-3p in an AGO2-dependent manner. In addition, the 3′-untranslated region of DAPK2 was fused to the luciferase coding region and transfected into HEK293 cells with miR-22-3p mimic, and the results showed that DAPK2 was a direct target of miR-22-3p. Our findings also indicated that MIAT overexpression could counteract the inhibitory effect of miR-22-3p on DAPK2. Moreover, MIAT knockdown was found to reduce DAPK2 expression and inhibit apoptosis in cardiomyocytes exposed to high glucose. In conclusion, our study demonstrates that MIAT may function as a competing endogenous RNA to upregulate DAPK2 expression by sponging miR-22-3p, which consequently leads to cardiomyocyte apoptosis involved in the pathogenesis of DCM.

Diabetic cardiomyopathy (DCM), which is defined as myocardial dysfunction occurring in the absence of coronary artery disease and hypertension, carries a substantial risk for the subsequent development of heart failure.^1^ There is growing evidence that oxidative stress, inflammation, mitochondrial dysfunction, impaired calcium handling, renin-angiotensin system activation, cardiomyocyte apoptosis are involved in the pathogenesis of DCM.^2^

Long non-coding RNAs (lncRNAs) represent a class of transcripts longer than 200 nucleotides without protein-coding function. They have critical roles in various biological processes, including gene expression regulation, genomic imprinting, nuclear-cytoplasmic trafficking, RNA splicing and translational control.^3^ We previously established a rat model of DCM and performed a microarray to identify the differentially expressed lncRNAs in myocardial tissue. We found that myocardial infarction–associated transcript (MIAT) was significantly upregulated in diabetic rats. MIAT has been identified as a risk allele for myocardial infarction in a large-scale case–control association study in Japanese subjects.^4^ A recent study revealed that MIAT might act as a competing endogenous RNA (ceRNA), and form a feedback loop with vascular endothelial growth factor and miR-150-5p to regulate endothelial cell function.^5^

Death-associated protein kinase 2 (DAPK2) belongs to a family of calmodulin-dependent serine/threonine kinases and has a key role in the regulation of cellular apoptosis.^6, 7^ The present study was designed to determine whether MIAT could modulate DAPK2 expression in the pathogenesis of DCM. Using *in silico* prediction and *in vitro* functional assays, we found that MIAT could function as a ceRNA to upregulate DAPK2 by sponging miR-22-3p, which consequently contributes to cardiomyocyte apoptosis in DCM.

## Results

### MIAT knockdown improves cardiac structure and function in DCM

**Figure 1:**
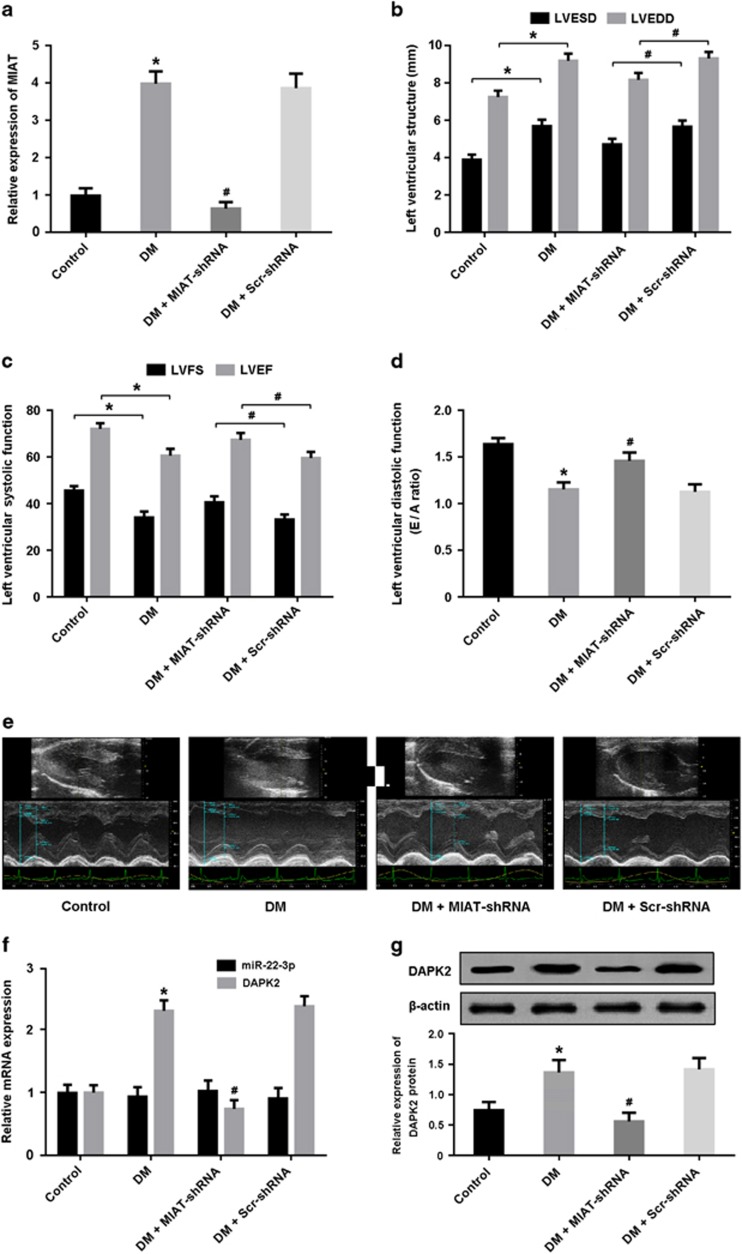
MIAT knockdown improves cardiac structure and function in diabetic rats. (**a**) The expression of MIAT in myocardium was detected by real-time PCR. (**b**–**e**) Left ventricular structure and function were assessed by echocardiography. (**f**,**g**) The expression of miR-22-3p and DAPK2 was analyzed by real-time PCR and western blot. *P<0.05 compared with control; ^#^*P*<0.05 compared with DM+Scr-shRNA (*n*=5 per group)

We generated the diabetic rat model and investigated the effects of MIAT knockdown on cardiac structure and function using echocardiography. The expression of MIAT was significantly upregulated in the myocardium of diabetic rats and downregulated after transfection with MIAT-shRNA (Figure 1a). The results of echocardiography showed that LVEDD and LVESD were remarkably increased in the DM group and decreased in the DM+MIAT-shRNA group (Figures 1b and e). In addition, LVFS, LVEF and E/A ratio, indicators of left ventricular systolic and diastolic function, were found to be reduced in diabetic rats, whereas MIAT knockdown could improve left ventricular dysfunction induced by hyperglycemia (Figures 1c and d). DAPK2, a kinase critically involved in the modulation of cellular apoptosis, was found to be upregulated in the myocardium of diabetic rats and downregulated after treatment with MIAT-shRNA (Figures 1f and g).

### MIAT knockdown attenuates myocardial hypertrophy and apoptosis in DCM

**Figure 2:**
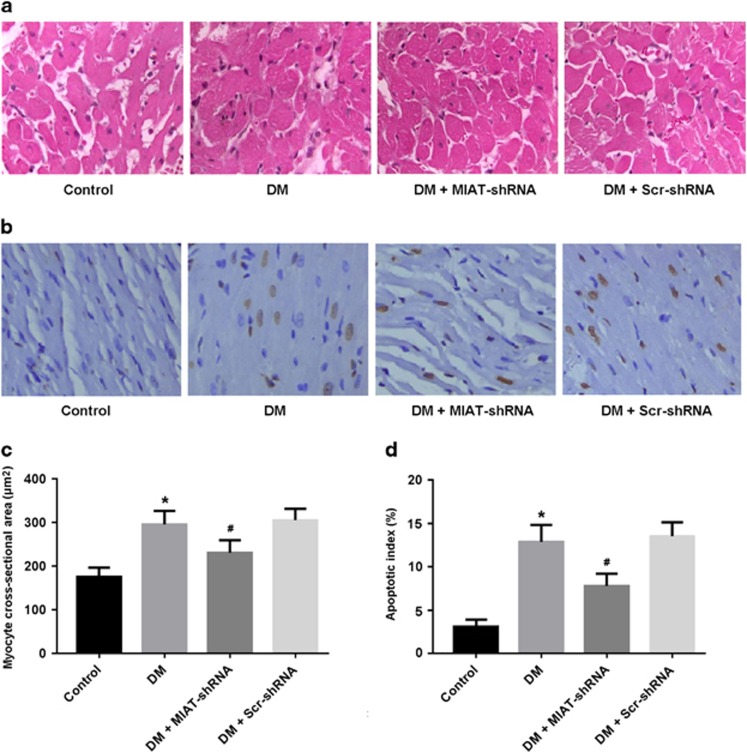
MIAT knockdown attenuates myocardial hypertrophy and apoptosis in diabetic rats. (**a**,**c**) Left ventricular tissue was stained with hematoxylin-eosin and myocyte cross-sectional area was calculated. (**b**,**d**) Cardiomyocyte apoptosis was determined by TUNEL staining and the apoptotic index was calculated. *P<0.05 compared with control; ^#^*P*<0.05 compared with DM+Scr-shRNA (*n*=5 per group)

Left ventricular tissue was stained with HE and MCSA was calculated to assess myocardial hypertrophy. Our results showed that MCSA was significantly increased in the DM group and decreased in the DM+MIAT-shRNA group (Figures 2a and c). Cellular apoptosis was detected by TUNEL staining and the apoptotic index was calculated. Our findings indicated that cardiomyocyte apoptosis was increased in diabetic rats, whereas MIAT knockdown could attenuate apoptosis induced by hyperglycemia (Figures 2b and d).

### MIAT is involved in the regulation of high glucose-induced apoptosis

**Figure 3:**
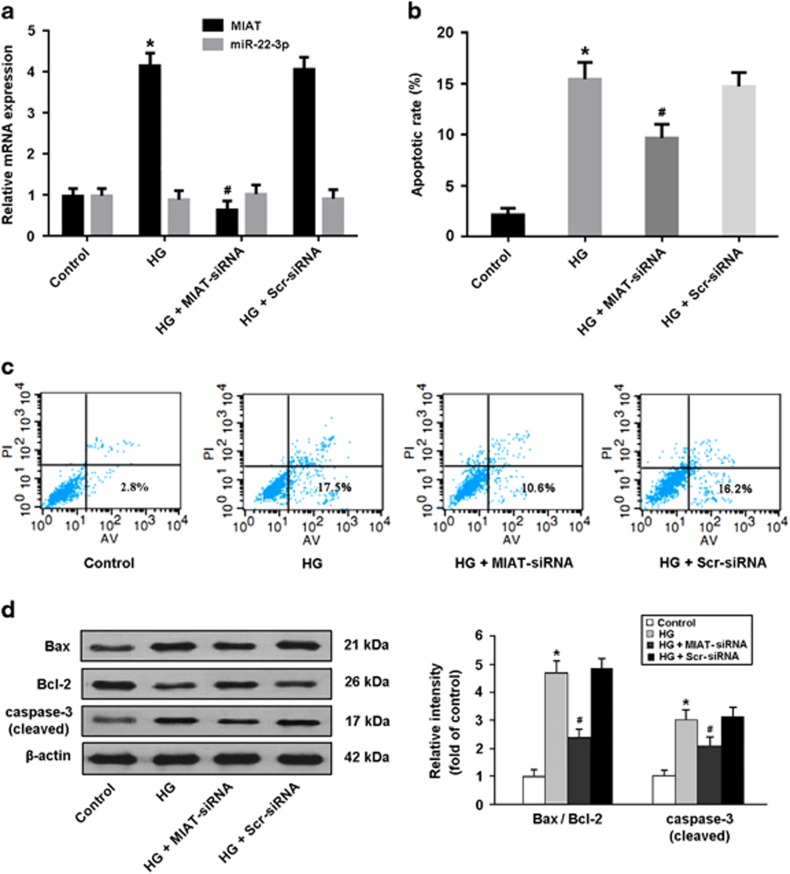
MIAT is involved in the regulation of high glucose-induced apoptosis. (**a**) Cardiomyocytes were transfected with adenoviral MIAT-siRNA or Scr-siRNA and then exposed to high glucose (HG, 30 mmol/l) for 48 h. The expression of MIAT and miR-22-3p was measured by real-time PCR. (**b**,**c**) Cardiomyocytes were stained with Annexin V/PI and then subjected to flow cytometry to detect apoptosis. (**d**) The expression of apoptosis-regulatory proteins was determined by Western blot. *P<0.05 compared with control; ^#^*P*<0.05 compared with HG+Scr-siRNA (*n*=5 per group)

Neonatal rat cardiomyocytes were transfected with MIAT-siRNA or Scr-siRNA before high glucose exposure and cellular apoptosis was evaluated by flow cytometry. The results showed that high glucose increased MIAT expression and induced cardiomyocyte apoptosis, whereas MIAT knockdown could suppress high glucose-mediated apoptosis (Figures 3a–c). Moreover, the cleaved caspase-3 expression and Bax/Bcl-2 ratio were found to be elevated in cardiomyocytes exposed to high glucose, while MIAT knockdown was associated with reduced expression of proapoptotic proteins (Figure 3d).

### MIAT is targeted by miR-22-3p in an AGO2-dependent manner

**Figure 4:**
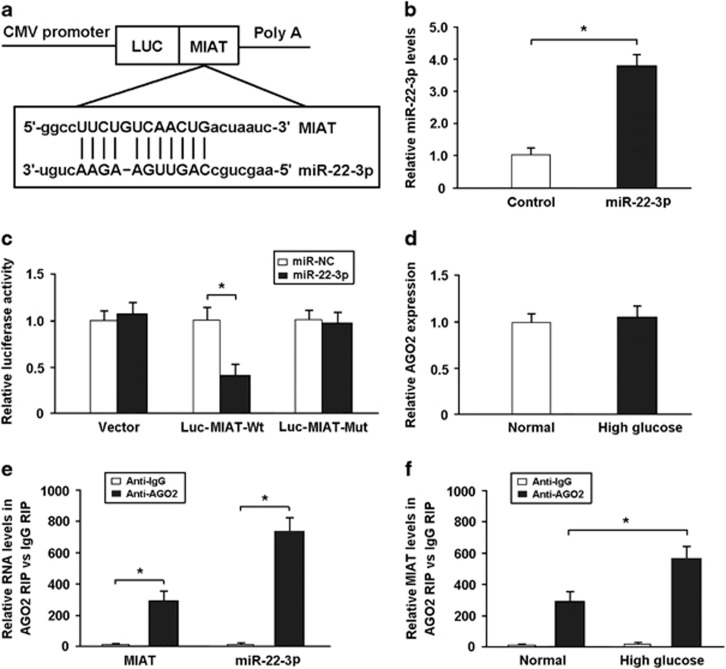
MIAT is targeted by miR-22-3p in an AGO2-dependent manner. (**a**) Bioinformatics prediction using miRcode indicated that MIAT sequence contained the putative binding site of miR-22-3p. (**b**) The miR-22-3p expression was increased in HEK293 cells transfected with miR-22-3p mimic. (**c**) The cDNA of MIAT was cloned into the downstream of luciferase gene (Luc-MIAT-Wt) and transfected into HEK293 cells with miR-22-3p mimic or control oligonucleotide. To avoid unspecific binding, the miR-22-3p binding sites in MIAT were mutated to generate Luc-MIAT-Mut. Luciferase activity was detected 48 h after transfection. (**d**) The AGO2 expression was detected in cardiomyocytes under basic conditions and high glucose exposure. (**e**) The RIP assay was performed to confirm whether MIAT and miR-22-3p could directly bind to AGO2 in cardiomyocytes. (**f**) The RIP experiments were conducted under basic conditions and high glucose exposure, and then the MIAT levels were determined. *P<0.05 (*n*=3 independent experiments)

Bioinformatics prediction using miRcode indicated that MIAT sequence contained the putative binding site of miR-22-3p (Figure 4a). The expression of miR-22-3p was increased in HEK293 cells transfected with miR-22-3p mimic (Figure 4b). The cDNA of MIAT was cloned into the downstream of luciferase gene (Luc-MIAT-Wt) and transfected into HEK293 cells with miR-22-3p mimic. To avoid unspecific binding, we also mutated the miR-22-3p binding site of MIAT to generate Luc-MIAT-Mut. The results of luciferase assay revealed that miR-22-3p transfection could reduce Luc-MIAT-Wt activity, but had no effect on Luc-MIAT-Mut activity (Figure 4c), which suggests that MIAT is the direct target of miR-22-3p.

miRNAs are present in the form of miRNA ribonucleoprotein complexes (miRNPs) which contains AGO2, a core component of the RNA-induced silencing complex. Our findings indicated that high glucose had no effect on the expression of AGO2 (Figure 4d). To determine whether MIAT associates with miRNPs, the RNA-binding protein immunoprecipitation (RIP) assay was performed in cardiomyocytes using AGO2 antibody. MIAT and miR-22-3p were found to be preferentially enriched in AGO2-containing miRNPs relative to IgG immunoprecipitates (Figure 4e). Moreover, the RIP experiments were carried out under basic conditions and high glucose exposure, and the results showed that MIAT could bind more to AGO2 under high glucose conditions. (Figure 4f).

### DAPK2 is a direct target of miR-22-3p in cardiomyocytes

**Figure 5:**
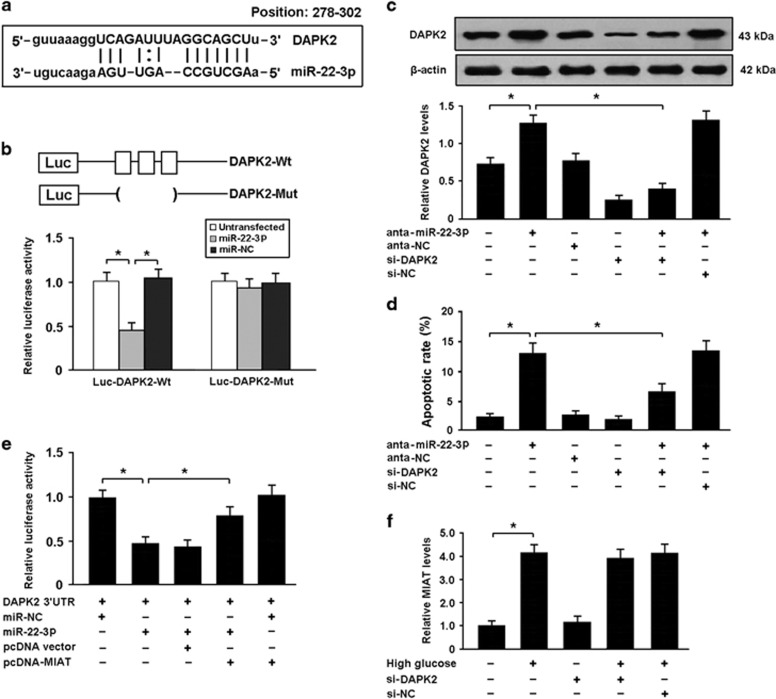
DAPK2 is a direct target of miR-22-3p in cardiomyocytes. (**a**) DAPK2 was predicted as a target gene of miR-22-3p using miRBase. (**b**) HEK293 cells were transfected with miR-22-3p mimic and luciferase constructs of DAPK2 3′-UTR (Luc-DAPK2-Wt) or mutant (Luc-DAPK2-Mut). Luciferase activity was detected 48 h after transfection. (**c**) Cardiomyocytes were transfected with miR-22-3p antagomir and/or adenoviral DAPK2 siRNA. The protein expression of DAPK2 was determined by western blot. (**d**) The apoptotic rate of cardiomyocytes was detected by flow cytometry following Annexin V/PI staining. (**e**) HEK293 cells were transfected with luciferase constructs of DAPK2 3′-UTR, miR-22-3p mimic and pcDNA-MIAT. The luciferase assay was performed to verify whether MIAT could competitively inhibit the binding of miR-22-3p to DAPK2. (**f**) Cardiomyocytes transfected with adenoviral DAPK2 siRNA or scramble siRNA were exposed to high glucose for 48 h and the MIAT expression was determined. *P<0.05 (*n*=3 independent experiments)

Among the putative targets of miR-22-3p, we focused on DAPK2, which is involved in the regulation of cellular apoptosis (Figure 5a). The 3′-untranslated region (UTR) of DAPK2 was fused to the luciferase coding region and transfected into HEK293 cells with miR-22-3p mimic. The luciferase assay revealed that DAPK2 was a direct target of miR-22-3p (Figure 5b). The expression of DAPK2 was upregulated in cardiomyocytes transfected with miR-22-3p antagomir, which consequently resulted in increased apoptosis. However, DAPK2 knockdown could reduce apoptosis in cardiomyocytes with miR-22-3p antagomir transfection, suggesting that miR-22-3p downregulation promotes apoptosis by increasing DAPK2 expression (Figures 5c and d).

To confirm whether MIAT competitively inhibits the binding of miR-22-3p to DAPK2, we conducted luciferase reporter assays in HEK293 cells. The results showed that MIAT could counteract the inhibitory effect of miR-22-3p on DAPK2 (Figure 5e). In addition, there were no significant changes in MIAT expression when DAPK2 was knockdown in cardiomyocytes exposed to high glucose (Figure 5f). Our data suggest that MIAT might act as a ceRNA to regulate DAPK2 expression by sponging miR-22-3p.

### High glucose induces apoptosis by regulating MIAT/DAPK2 pathway

**Figure 6:**
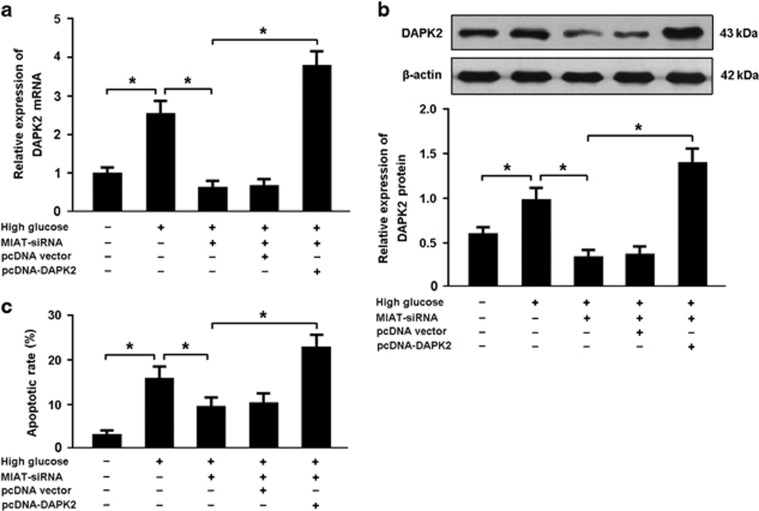
High glucose induces apoptosis by regulating MIAT/DAPK2 pathway. (**a**,**b**) Cardiomyocytes were transfected with MIAT-siRNA and/or pcDNA-DAPK2 before high glucose (30 mmol/l) exposure and the expression of DAPK2 was analyzed by real-time PCR and western blot. (**c**) Cardiomyocytes were stained with Annexin V/PI and the apoptotic rate was determined by flow cytometry. *P<0.05. (*n*=3 independent experiments)

Cardiomyocytes were transfected with MIAT-siRNA and/or pcDNA-DAPK2 prior to exposure to high glucose and cellular apoptosis was evaluated by flow cytometry. Our results indicated that high glucose was associated with increased DAPK2 expression and cardiomyocyte apoptosis, whereas MIAT knockdown reduced DAPK2 expression and inhibited apoptosis in high glucose-exposed cardiomyocytes. However, MIAT-siRNA and pcDNA-DAPK2 cotransfection could promote apoptosis in cardiomyocytes exposed to high glucose (Figures 6a–c).

## Discussion

It has been well documented that lncRNAs have critical roles in the regulation of various cardiovascular diseases, but little is known about the function of lncRNAs in the pathogenesis of DCM, an important cardiovascular complication of diabetes. In this study, we established a streptozocin-induced diabetic rat model to investigate the pathologic role of lncRNA MIAT in the development of DCM. Our results showed that MIAT was significantly upregulated in the myocardium of diabetic rats, and MIAT knockdown was found to alleviate cardiomyocyte apoptosis and improve left ventricular function. We then further explored the molecular mechanisms underlying the involvement of MIAT in high glucose-induced apoptosis. Our findings demonstrated that MIAT could function as a ceRNA to upregulate DAPK2 expression by sponging miR-22-3p, which consequently results in cardiomyocyte apoptosis involved in the progression of DCM.

The lncRNA MIAT, also termed as Gomafu, was first identified to be associated with myocardial infarction in a genome-wide association study.^4^ Increasing evidence has suggested that MIAT is involved in various diseases and cellular processes, including myocardial infarction,^8^ microvascular dysfunction,^5^ age-related cataract,^9^ and neurogenic commitment.^10^ Recently, Crea *et al.*^11^ reported that MIAT could be exploited as a new biomarker and therapeutic target for neuroendocrine prostate cancer. In addition, Ip *et al.*^12^ showed that MIAT knockout mice exhibited hyperactive behaviors and enhanced responsiveness to the psychostimulant methamphetamine. In this study, our findings indicated that high glucose could upregulate the expression of MIAT, which consequently leads to increased cardiomyocyte apoptosis.

The ceRNA hypothesis has been proposed that protein-coding messenger RNAs and non-coding RNAs communicate with each other by competing for binding to shared miRNAs, a family of small non-coding RNAs responsible for the post-transcriptional regulation of gene expression.^13^ Understanding this novel RNA cross-talk may provide valuable insights into the gene regulatory networks involved in the pathogenesis of cardiovascular diseases. In the present study, using bioinformatics prediction and functional assays, we found that lncRNA MIAT could directly bind to miR-22-3p and function as a ceRNA to regulate the expression of DAPK2 in cardiomyocytes, which might be an important molecular mechanism involved in high glucose-induced apoptosis.

Apoptosis, which is characterized by cell shrinkage, plasma membrane blebbing, chromatin compaction and nuclear fragmentation, has been shown to participate in the pathogenesis of DCM.^2^ Recently, Li *et al.*^14^ demonstrated that H19/miR-675 axis was involved in the modulation of hyperglycemia-induced apoptosis by targeting VDAC1, which may provide a novel therapeutic strategy for DCM. In addition, downregulation of MALAT1 was found to reduce cardiomyocyte apoptosis and improve left ventricular function in diabetic rats.^15^ In the present study, our findings revealed that MIAT could promote cardiomyocyte apoptosis by increasing DAPK2 expression in DCM. When silencing miR-22-3p, there was still a significant induction of apoptosis after DAPK2 knockdown, suggesting that DAPK2 is not the only mediator of apoptosis targeted by miR-22-3p. DAPK2 belongs to the DAPK family, which consists of a number of serine/threonine kinases regulated by calcium/calmodulin that are involved in death-inducing pathways. MIAT upregulation may become a sink for miR-22-3p, thereby affecting the derepression of DAPK2. The ceRNA regulatory network, MIAT/miR-22-3p/DAPK2, will provide a novel insight into the mechanism of high glucose-induced cardiomyocyte apoptosis.

In summary, our study demonstrates that lncRNA MIAT is upregulated in the myocardium of diabetic rats and may act as a ceRNA to increase DAPK2 expression by sponging miR-22-3p, which consequently contributes to cardiomyocyte apoptosis involved in the pathogenesis of DCM.

## Materials and methods

### Animal model and treatment

All experiments were performed in accordance with the Guide for the Care and Use of Laboratory Animals and were approved by the Animal Ethics Committee of Soochow University. Male Sprague–Dawley rats weighing 200–250 g were obtained from the Experimental Animal Center of Soochow University. The diabetic rat model was induced by a single intraperitoneal injection of streptozotocin (65 mg/kg, Sigma, St. Louis, MO, USA) as previously described.^16^ Diabetic rats were intracoronarily administered 80 *μ*l lentivirus MIAT-shRNA (DM+MIAT-shRNA) or scramble shRNA (DM+Scr-shRNA), and were then kept for 12 weeks together with the normal rats (Control) and the diabetic rats without lentivirus administration (DM). The intracoronary delivery of lentivirus with aortic cross-clamping was described in detail by del Monte F and Hajjar RJ.^17^

### Cardiomyocyte culture and treatment

The neonatal rats were deeply anaesthetized with 1.0% isoflurane, and the hearts were surgically removed, washed instantly in cold D-Hanks solution and minced into 1–3 mm^3^ pieces. Cardiac tissues were dispersed in a series of incubations at 37 °C in D-Hanks solution containing 1.2 mg/ml pancreatin and 0.14 mg/ml collagenase (Gibco, Grand Island, NY, USA). After centrifugation, the cells were suspended in Dulbecco's modified Eagle's medium (Gibco) containing 20% calf serum, 100 U/ml penicillin and 100 *μ*g/ml streptomycin. The dissociated cells were preplated at 37 °C for 1 h to separate cardiomyocytes by adherence of cardiac fibroblasts. Thereafter, cells were collected and diluted to 1 × 10^6^ cells/ml and plated onto 1% gelatin-coated culture dishes.

### Echocardiographic study

Cardiac structure and function were assessed by two-dimensional echocardiography performed as previously described.^18^ The following measurements were obtained: left ventricular end-diastolic diameter (LVEDD), left ventricular end-systolic diameter (LVESD), left ventricular fractional shortening (LVFS), left ventricular ejection fraction (LVEF), and E wave-to-A wave ratio (E/A ratio). All measurements were averaged for three consecutive cardiac cycles.

### Histological analysis

Rat myocardial tissue was fixed in 10% buffered formalin, embedded in paraffin, and sliced into 5-*μ*m-thick sections. The slides were then stained with hematoxylin-eosin (HE) and observed under a light microscope. Myocyte cross-sectional area (MCSA) was measured using an image analysis software (Image Pro-Plus, Media Cybernetics, Silver Spring, MD, USA).

### TUNEL staining

Cardiomyocyte apoptosis was detected with the terminal deoxynucleotidyl transferase-mediated dUTP nick-end labeling (TUNEL) *in situ* cell death detection kit (Boehringer, Mannheim, Germany). The apoptotic index was calculated as the percentage of TUNEL-positive cells divided by the total number of cells. At least 10 representative fields were evaluated for each group and the average value was calculated.

### Annexin V-FITC /PI staining

Cardiomyocytes were stained with FITC-labeled Annexin V and propidium iodide (PI) (BD Pharmingen, Franklin Lakes, NJ, USA) and then subjected to flow cytometry to determine apoptosis. The apoptotic rate was calculated as the percentage of Annexin V-positive and PI-negative cells divided by the total number of cells in the gated region.

### Luciferase reporter assay

To verify whether DAPK2 was a direct target of miR-22-3p, we carried out luciferase experiments in HEK293 cells. The 3′-UTR of DAPK2 was cloned into the downstream of luciferase gene to generate Luc-DAPK2-Wt vector. The 3′-UTR without predicted miR-22-3p binding site was constructed to generate Luc-DAPK2-Mut vector. For luciferase assay, cells were plated in 24-well culture plates, and then transfected with either wild-type or mutant construct with and without miRNA mimic or negative control. Luciferase activity was measured 48 h after transfection using the Dual Luciferase Reporter Assay System (Promega, Madison, WI, USA).

### RNA-binding protein immunoprecipitation

The RIP assay was performed using the EZ-Magna RIP Kit (Millipore, Billerica, MA, USA). Briefly, cardiomyocytes were lysed in RIP lysis buffer, following incubation with RIP buffer containing magnetic beads conjugated with anti-AGO2 antibody (Millipore, USA) or negative control IgG. Anti-SNRNP70 was used as a positive control for the RIP procedure. The samples were incubated with proteinase K and immunoprecipitated RNAs were isolated. The precipitated RNAs were purified and subjected to quantitative PCR to detect the presence of target sequences.

### Real-time PCR

Total RNA was isolated from cardiac tissue and cells using TRIzol Reagent (Invitrogen, Carlsbad, CA, USA). RNA was reverse transcribed using SuperScript First Strand cDNA System (Invitrogen, USA). Quantitative PCR was performed using SYBR Green Taq ReadyMix (Sigma, USA) on an Applied Biosystems 7500 PCR system. GAPDH was used as the endogenous control in this study. The primer sequences are as follows: MIAT, 5′-GAGGGAAGTTCTGAGCTTGG-3′ and 5′-CCTTTCTTCTGGGCTGAGAC-3′ DAPK2, 5′-TCCTGGATGGGGTGAACTAC-3′ and 5′-CAGCTTGATGTGTGGAATGG-3′ GAPDH, 5′-TGCCCAGAACATCATCCCT-3′ and 5′-GGTCCTCAGTGTAGCCCAAG-3′. The relative expression of genes was presented as fold change and calculated using the 2^−ΔΔCT^ method.

### Western blotting

Equal amounts of protein were separated by SDS/PAGE (10% gel), transferred onto nitrocellulose membranes and blocked with 5% non-fat milk. The membranes were incubated with primary antibodies at 4 °C overnight, and then with horseradish peroxidase-conjugated secondary antibodies at room temperature for 1 h. The antibodies were purchased from Cell Signaling Technology and applied following the manufacturer’s instructions. The immunocomplexes were visualized with an enhanced chemiluminescence detection kit (Amersham Biosciences, Uppsala, Sweden).

### Statistical analysis

The data are expressed as mean±S.D., and the differences between groups were compared using one-way ANOVA with SPSS version 18.0. The Scheffé *post hoc* test was used for multiple comparisons if the ANOVA was significant. A value of *P*<0.05 was considered statistically significant.
